# Maximum Betti Numbers of Čech Complexes

**DOI:** 10.1007/s00454-025-00796-5

**Published:** 2025-11-10

**Authors:** Herbert Edelsbrunner, János Pach

**Affiliations:** 1 ISTA (Institute of Science and Technology Austria), Klosterneuburg, Austria; 2 Rényi Institute of Mathematics, Budapest, Hungary

**Keywords:** Discrete geometry, Computational topology, Čech complexes, Delaunay mosaics, Alpha complexes, Betti numbers, Extremal questions, 05B45, 52D17, 55N31

## Abstract

The Upper Bound Theorem for convex polytopes implies that the *p*-th Betti number of the Čech complex of any set of *N* points in ${{\mathbb R}}^d$ and any radius satisfies ${\beta }_{p}{} = O(N^{m})$, with $m = \min \{ p+1, {\big \lceil d/2 \big \rceil } \}$. We construct sets in even and odd dimensions that prove this upper bound is asymptotically tight. For example, we describe a set of $N = 2(n+1)$ points in ${{\mathbb R}}^3$ and two radii such that the first Betti number of the Čech complex at one radius is $(n+1)^2 - 1$, and the second Betti number of the Čech complex at the other radius is $n^2$.

## Introduction

Given a finite set of points $A \subseteq {{\mathbb R}}^d$ and a radius $r \ge 0$, the *Čech complex* of *A* and *r* consists of all subsets $B \subseteq A$ for which the intersection of the closed balls of radius *r* centered at the points in *B* is non-empty. This is an abstract simplicial complex isomorphic to the nerve of the balls, and by the Nerve Theorem [5], it has the same homotopy type as the union of the balls. This property is the reason for the popularity of the Čech complex in topological data analysis; see e.g. [7, 9]. Of particular interest are the *Betti numbers* of the union of balls, which may be interpreted as the numbers of holes of different dimensions. These are intrinsic properties, but for a space embedded in ${{\mathbb R}}^d$, they describe the connectivity of the space as well as that of its complement. Most notably, the (reduced) zero-th Betti number, ${\beta }_{0}{}$, is one less than the number of *connected components*, and the last possibly non-zero Betti number, ${\beta }_{d-1}{}$, is the number of *voids* (bounded components of the complement). Spaces that have the same homotopy type—such as a union of balls and the corresponding Čech complex—have identical Betti numbers. While the Čech complex is not necessarily embedded in ${{\mathbb R}}^d$, the corresponding union of balls is, which implies that also the Čech complex has no non-zero Betti numbers beyond dimension $d-1$. To gain insight into the statistical behavior of the Betti numbers of Čech complexes, it is useful to understand how large the numbers can get, and this is the question we study in this paper.

The question of maximum Betti numbers lies at the crossroads of computational topology and discrete geometry. Originally inspired by problems in the theory of polytopes [19, 27], optimization [22], robotics, motion planning [23], and molecular modeling [20], many interesting and surprisingly difficult questions were asked about the complexity of the union of *n* geometric objects, as *n* tends to infinity. For a survey, consult [1]. Particular attention was given to estimating the number of voids among *N* simply shaped bodies, e.g., for the translates of a fixed convex body in ${{\mathbb R}}^d$. In the plane, the answer is typically linear in *N* (for instance, for disks or other fat objects), but for $d=3$, the situation is more delicate. The maximum number of voids among *N* translates of a convex polytope with a constant number of faces is $\Theta (N^2)$, but this number reduces to linear for the cube and other simple shapes [3]. It was conjectured for a long time that similar bounds hold for the translates of a convex shape that is not necessarily a polytope. However, this turned out to be false: Aronov, Cheung, Dobbins and Goaoc [2] constructed a convex body in ${{\mathbb R}}^3$ for which the number of voids is $\Omega (N^3)$. This is the largest possible order of magnitude for any arrangement of convex bodies, even if they are not translates of a fixed one [18]. It is an outstanding open problem whether there exists a *centrally symmetric* convex body with this property.

For the special case where the convex body is the *unit ball* in ${{\mathbb R}}^3$, the maximum number of voids in a union of *N* translates is $O(N^2)$. This can be easily derived from the Upper Bound Theorem for 4-dimensional convex polytopes. It has been open for a long time whether this bound can be attained. Our main theorem answers this question in the affirmative, in a more general sense.

### MainTheorem

For every $d \ge 1$, $0 \le p \le d-1$, and $N \ge 1$, there is a set of *N* points in ${{\mathbb R}}^d$ and a radius such that the *p*-th Betti number of the Čech complex of the points and the radius is ${\beta }_{p}{} = \Theta (N^m)$, with $m = \min \{ p+1, {\big \lceil d/2 \big \rceil } \}$.

For $d=3$, the maximum second Betti number is ${\beta }_{2}{} = \Theta (N^2)$, which is equivalent to the maximum number of voids being $\Theta (N^2)$. In addition to the Čech complex, the proof of the Main Theorem makes use of three complexes defined for a set of *N* points, $A \subseteq {{\mathbb R}}^d$, in which the third also depends on a radius $r \ge 0$:

- the *Voronoi domain* of a point $a \in A$, denoted ${\textrm{dom}{({a},{A})}}$, contains all points $x \in {{\mathbb R}}^d$ that are at least as close to *a* as to any other point in *A*, and the *Voronoi tessellation* of *A*, denoted ${\textrm{Vor}{({A})}}$, is the collection of domains ${\textrm{dom}{({a},{A})}}$ with $a \in A$ [25];
- the *Delaunay mosaic* of *A*, denoted ${\textrm{Del}{({A})}}$, contains the convex hull of $\Sigma \subseteq A$ if the common intersection of the ${\textrm{dom}{({a},{A})}}$, with $a \in \Sigma$, is non-empty, and no other Voronoi domain contains this common intersection [8]; it is closed under taking faces and therefore is a polyhedral complex;
- the *Alpha complex* of *A* and *r*, denoted ${\textrm{Alf}{({A},{r})}}$, is the subcomplex of the Delaunay mosaic that contains the convex hull of $\Sigma$ if the common intersection of the ${\textrm{dom}{({a},{A})}}$, with $a \in \Sigma$, contains a point at distance at most *r* from the points in $\Sigma$; see [10, 11]. If a cell in ${\textrm{Del}{({A})}}$ satisfies this property, then all its faces satisfy the property, which implies that ${\textrm{Alf}{({r},{A})}}$ is a complex, and thus indeed a subcomplex of ${\textrm{Del}{({A})}}$.

The Delaunay mosaic is also known as the *dual* of the Voronoi tessellation, or the *Delaunay triangulation* of *A*. Note that ${\textrm{Alf}{({A},{r})}} \subseteq {\textrm{Alf}{({A},{R})}}$ whenever $r \le R$, and that for sufficiently large radius, the Alpha complex is the Delaunay mosaic. Similar to the Čech complex, the Alpha complex has the same homotopy type as the union of balls with radius *r* centered at the points in *A*, and thus the same Betti numbers. It is instructive to increase *r* from 0 to $\infty$ and to consider the *filtration* or nested sequence of Alpha complexes. The difference between an Alpha complex, *K*, and the next Alpha complex in the filtration, *L*, consists of one or more cells. If it is a single cell of dimension *p*, then either ${\beta }_{p}{} (L) = {\beta }_{p}{} (K) + 1$ or ${\beta }_{p-1}{} (L) = {\beta }_{p-1}{} (K) - 1$, and all other Betti numbers are the same. In the first case, we say the cell gives *birth* to a *p*-cycle, while in the second case, it gives *death* to a $(p-1)$-cycle, and in both cases we say it is *critical*. If there are two or more cells in the difference, this may be a generic event or accidental due to non-generic position of the points. In the simplest generic case, we simultaneously add two cells (one a face of the other), and the addition is an anti-collapse, which does not affect the homotopy type of the complex. More elaborate anti-collapses, such as the simultaneous addition of an edge, two triangles, and a tetrahedron, can arise generically. The cells in an interval of size 2 or larger cancel each other’s effect on the homotopy type, so we say these cells are *non-critical*. We refer to [4] for more details.

With these notions, it is not difficult to prove the upper bounds in the Main Theorem. As mentioned above, the Čech and alpha complexes for radius *r* have the same Betti numbers. Since a *p*-cycle is given birth to by a *p*-cell in the filtration of Alpha complexes, and every *p*-cell gives birth to at most one *p*-cycle, the number of *p*-cells is an upper bound on the number of *p*-cycles, which are counted by the *p*-th Betti number. The number of *p*-cells in the Alpha complex is at most that number in the Delaunay mosaic, which, by the Upper Bound Theorem for convex polytopes [19, 27], is at most $O(N^m)$, with $m = \min \{p+1, {\big \lceil d/2 \big \rceil }\}$.

By comparison, to come up with constructions that prove matching lower bounds is delicate and the main contribution of this paper. Our constructions are multipartite and inspired by Lenz’ constructions related to Erdős’s celebrated question on repeated distances [13]: “what is the largest number of point pairs $\{ a,b \}$ in an *N*-element set in ${{\mathbb R}}^d$ with ${\Vert {a}-{b}\Vert } = 1$?” Lenz noticed that in 4 (and higher) dimensions, this maximum is $\Theta (N^2)$. To see this, take two circles of radius  centered at the origin, lying in two orthogonal planes, and place $\lceil N/2\rceil$ and $\lfloor N/2\rfloor$ points on them. By Pythagoras’ theorem, the distance between any two points on different circles is 1, so the number of unit distances is roughly $N^2/4$, which is nearly optimal. For $d=2$ and 3, we are far from knowing asymptotically tight bounds. The current best constructions give $\Omega (N^{1+c/\log \log N})$ unit distance pairs in the plane [6, page 191] and $\Omega (N^{4/3}\log \log N)$ in ${{\mathbb R}}^3$, while the corresponding upper bounds are $O(N^{4/3})$ and $O(N^{3/2})$; see [24] and [17, 26]. Even the following, potentially simpler, bipartite repeated distance question is open in ${{\mathbb R}}^3$: “given *N* red points and *N* blue points in ${{\mathbb R}}^3$, such that the minimum distance between a red and a blue point is 1, what is the largest number of red-blue point pairs that determine a unit distance?” The best known upper bound, due to Edelsbrunner and Sharir [12] is $O(N^{4/3})$, but we have no superlinear lower bound. This last question is closely related to the subject of our present paper.

It is not difficult to see that the upper bounds in the Main Theorem also hold for the Betti numbers of the union of *N* *not necessarily congruent* balls in ${{\mathbb R}}^d$. This requires the use of weighted versions of the Voronoi tessellation and the Upper Bound Theorem. In the lower bound constructions, much of the difficulty stems from the fact that we insist on using congruent balls. This suggests the analogy to the problem of repeated distances.

**Outline.** Section 2 proves the Main Theorem for sets in *even* dimensions. Starting with Lenz’ constructions, we partition the Delaunay mosaic into finitely many groups of *congruent* simplices. We compute the radii of their circumspheres and obtain the Betti numbers by straightforward counting. In Section 3, we establish the Main Theorem for sets in three dimensions. The situation is more delicate now, because the simplices of the Delaunay mosaic no longer fall into a small number of distinct congruence classes. Nevertheless, they can be divided into groups of nearly congruent simplices, which will be sufficient to carry out the counting argument. In Section 4, we extend the result to any *odd* dimension. Again we require a detailed analysis of the shapes and sizes of the simplices, which now proceeds by induction on the dimension. Section 5 contains concluding remarks and open questions.

## Even Dimensions

In this section, we give an answer to the maximum Betti number question for Čech complexes in even dimensions. To state the result, let $n_k$ be the minimum integer such that the edges of a regular $n_k$-gon inscribed in a circle of radius $\sqrt{2}/2$ are strictly shorter than $\sqrt{2/k}$. For $k=1$ we have $n_1 = 3$, and for $k=2$ we have $n_2=5$, because the side length of an inscribed square is equal to 1.

### Theorem 2.1

(Maximum Betti Numbers in ${{\mathbb R}}^{2k}$) For every $2k \ge 2$ and $n \ge n_k$, there exist a set *A* of $N = kn$ points in ${{\mathbb R}}^{2k}$ and radii $\rho _0< \rho _1< \ldots < \rho _{2k-2}$ such that12For $p = 2k-1$, there exist $N = k(n+1) + 2$ points in ${{\mathbb R}}^{2k}$ and a radius such that the *p*-th Betti number of the Čech complex is $n^k \pm O(n^{k-1})$.

The reason for the condition $n \ge n_k$ will become clear in the proof of Lemma 2.5, which establishes a particular ordering of the circumradii of the cells in the Delaunay mosaic. The proof of the cases $0 \le p \le 2k-2$ is not difficult and uses elementary computations, the results of which will be instrumental for establishing the more challenging odd-dimensional statements in Sections 3 and 4. The proof consists of four steps presented in four subsections: the construction of the point set in Section 2.1, the geometric analysis of the simplices in the Delaunay mosaic in Section 2.2, the ordering of the circumradii in Section 2.3, and the final counting in Section 2.4. The proof of the case $p = 2k-1$ in ${{\mathbb R}}^{2k}$ readily follows the case $p = 2k-2$ in ${{\mathbb R}}^{2k-1}$, as we will explain in Section 4.6.

### Construction

Let $d = 2k$. We construct a set $A = A_{2k} (n)$ of $N = kn$ points in ${{\mathbb R}}^d$ using *k* concentric circles in mutually orthogonal coordinate planes: for $0 \le \ell \le k-1$, the circle ${{C}_{\ell }}$ with center at the origin, $0 \in {{\mathbb R}}^{d}$, is defined by $x_{2 \ell + 1}^2 + x_{2 \ell +2}^2 = \frac{1}{2}$ and $x_i = 0$ for all $i \ne 2\ell +1, 2\ell +2$. On each of the *k* circles, we choose $n \ge 3$ points that form a regular *n*-gon. The length of the edges of these *n*-gons will be denoted by 2*s*. Obviously, we have $s = \frac{\sqrt{2}}{2} \sin \frac{\pi }{n}$. Assuming $k \ge 2$, the condition $n \ge n_k$ implies that the Euclidean distance between consecutive points along the same circle is less than 1, and by Pythagoras’ theorem, the distance between any two points on different circles is 1. It follows that for $r = \frac{1}{2}$, neighboring balls centered on the same circle overlap, while the balls centered on different circles only touch. Correspondingly, the first Betti number of the Čech complex for a radius slightly less than $\frac{1}{2}$ is ${\beta }_{1}{} = k$. To get the first Betti number for $r = \frac{1}{2}$, we add all edges of length 1, of which $k-1$ connect the *k* circles into a single connected component, while the others increase the first Betti number to $${\beta }_{1}{} = k + \left( {\begin{array}{c}k\\ 2\end{array}}\right) n^2 - (k-1) = \left( {\begin{array}{c}k\\ 2\end{array}}\right) n^2 + 1$$.

To generalize the analysis beyond the first Betti number, we consider the Delaunay mosaic and two radii defined for each of its cells. The *circumsphere* of a *p*-cell is the unique $(p-1)$-sphere that passes through its vertices, and we call its center and radius the *circumcenter* and the *circumradius* of the cell. To define the second radius, we call a $(d-1)$-sphere *empty* if all points of *A* lie on or outside the sphere. The *radius function* on the Delaunay mosaic, ${\textrm{Rad}}:{\textrm{Del}{({A})}} \rightarrow {{\mathbb R}}$, maps each cell to the radius of the smallest empty $(d-1)$-sphere that passes through the vertices of the cell. By construction, each Alpha complex is a sublevel set of this function: ${\textrm{Alf}{({A},{r})}} = {\textrm{Rad}}^{-1} [0,r]$. The two radii of a cell may be different, but they agree for the critical cells as defined in terms of their topological effect in the introduction. It will be convenient to work with the corresponding geometric characterization of criticality:

#### Definition 2.2

*(Critical Cell)* A *critical cell* of ${\textrm{Rad}}:{\textrm{Del}{({A})}} \rightarrow {{\mathbb R}}$ is a cell $\Sigma \in {\textrm{Del}{({A})}}$ that (1) contains the circumcenter in its interior, and (2) the $(d-1)$-sphere centered at the circumcenter that passes through the vertices of $\Sigma$ is empty and the vertices of $\Sigma$ are the only points of *A* on this sphere.

There are two conditions for a cell to be critical for a reason. The first guarantees that its topological effect is not canceled by one of its faces, and the second guarantees that it does not cancel the topological effect of one of the cells it is a face of. As proved in [4], the radius function of a generic set, $A \subseteq {{\mathbb R}}^d$, is *generalized discrete Morse*; see Forman [14] for background on discrete Morse functions. This means that each level set of ${\textrm{Rad}}$ is a union of disjoint combinatorial intervals, and a simplex is critical iff it is the only simplex in its interval. Our set *A* is not generic because the $(d-1)$-sphere with center $0 \in {{\mathbb R}}^{2k}$ and radius  passes through all its points. Indeed, ${\textrm{Del}{({A})}}$ is really a 2*k*-dimensional convex polytope, namely the convex hull of *A* and all its faces. Nevertheless, the distinction between critical and non-critical cells is still meaningful, and all cells in the Delaunay mosaic of our construction will be seen to be critical.

The value of the 2*k*-polytope under the radius function is , while the values of its proper faces are strictly smaller than . Let ${\Sigma }_{\ell ,j}$ be such a face, in which $\ell +1$ is the number of circles that contain one or two of its vertices, and $j+1$ is the number of circles that contain two. This face is a simplex of dimension $\mathrm{dim\,}{{\Sigma }_{\ell ,j}} = \ell + 1 + j$, and it has $j+1$ disjoint *short* edges of length 2*s*, while the remaining *long* edges all have unit length. Indeed, the geometry of the simplex is determined by $\ell$ and *j* and does not depend on the circles from which we pick the vertices or where along these circles we pick them, as long as two vertices from the same circle are consecutive along this circle. For example, ${\Sigma }_{1,-1}$, ${\Sigma }_{1,0}$, and ${\Sigma }_{1,1}$ are the unit length edge, the isosceles triangle with one short and two long edges, and the tetrahedron with two disjoint short and four long edges, respectively. We call the ${\Sigma }_{\ell ,j}$ *ideal simplices*. In even dimensions they are *precisely* the simplices in the Delaunay mosaic of our construction. However, in odd dimensions, the cells in the Delaunay mosaic only *converge* to the ideal simplices. This will be explained in detail in Sections 3 and 4.

### Circumradii of Ideal Simplices

In this section, we compute the sizes of some ideal simplices, beginning in four dimensions. The *ideal 2-simplex* or *triangle*, denoted ${\Sigma }_{1,0}$, is the isosceles triangle with one short and two long edges. We write *h*(*s*) for the *height* of ${\Sigma }_{1,0}$ (the distance between the midpoint of the short edge and the opposite vertex), and *r*(*s*) for the circumradius. There is a unique way to glue four such triangles to form the boundary of a tetrahedron: the two short edges are disjoint and their endpoints are connected by four long edges. This is the *ideal 3-simplex* or *tetrahedron*, denoted ${\Sigma }_{1,1}$. We write *H*(*s*) for its *height* (the distance between the midpoints of the two short edges), and *R*(*s*) for its circumradius.

#### Lemma 2.3

(Ideal Triangle and Tetrahedron) The squared heights and circumradii of the ideal triangle and the ideal tetrahedron in ${{\mathbb R}}^4$ satisfy

3 $$\begin{aligned} h^2(s)&= 1 - s^2 , ~~~~~~~~ 4r^2(s) = \frac{1}{1-s^2} , \end{aligned}$$

4 $$\begin{aligned} H^2(s)&= 1 - 2s^2, ~~~~~~ 4R^2(s) = 1 + 2s^2 . \end{aligned}$$

#### Proof

By Pythagoras’ theorem, the squared height of the ideal triangle is $h^2 = 1-s^2$. If we glue the two halves of a scaled copy of the ideal triangle to the two halves of the short edge, we get a quadrangle inscribed in the circumcircle of the triangle. One of its diagonals passes through the center, and its squared length satisfies $4r^2 = 1 + (s/h)^2 = 1 + \frac{s^2}{1-s^2}$.

By Pythagoras’ theorem, the squared height of the ideal tetrahedron is $H^2 = h^2 - s^2 = 1 - 2 s^2$. Hence, the squared diameter of the circumsphere is $4R^2 = H^2 + (2s)^2 = 1+2s^2$. $\square$

To generalize the analysis beyond the ideal simplices in four dimensions, we write  for the circumradius of ${\Sigma }_{\ell ,j}$, so , , and . For two kinds of ideal simplices, the circumradii are particularly easy to compute, namely for the ${\Sigma }_{\ell ,-1}$ and the ${\Sigma }_{\ell ,\ell }$, and we will see that knowing their circumradii will be sufficient for our purposes.

#### Lemma 2.4

(Further Ideal Simplices) For $\ell \ge 0$, the squared circumradii of ${\Sigma }_{\ell ,-1}$ and ${\Sigma }_{\ell ,\ell }$ satisfy ${r^{2}_{{\ell },{-1}}} (s) = {\ell }/{(2\ell +2)}$ and ${r^{2}_{{\ell },{\ell }}} (s) = {(\ell +2s^2)}/{(2\ell +2)}$.

#### Proof

Consider the standard $\ell$-simplex, which is the convex hull of the endpoints of the $\ell +1$ unit coordinate vectors in ${{\mathbb R}}^{\ell +1}$. Its squared circumradius is the squared distance between the barycenter and any one of the vertices, which is easy to compute. By comparison, the squared circumradius of the regular $\ell$-simplex with unit length edges is half that of the standard $\ell$-simplex:

5 $$\begin{aligned} R_\ell ^2&= \frac{1}{2} \left[ \frac{\ell ^2}{(\ell +1)^2} + \frac{1}{(\ell +1)^2} + \ldots + \frac{1}{(\ell +1)^2} \right] = \frac{\ell }{2(\ell +1)} , \end{aligned}$$

Since ${r^{2}_{{\ell },{-1}}} (s) = R_\ell ^2$, this proves the first equation in the lemma. Note that the convex hull of the midpoints of the $\ell +1$ short edges of ${\Sigma }_{\ell ,\ell }$ is a regular $\ell$-simplex with edges of squared length $H^2(s) = 1-2s^2$. The short edges are orthogonal to this $\ell$-simplex, which implies

6 $$\begin{aligned} {r^{2}_{{\ell },{\ell }}}&= H^2(s) \cdot R_\ell ^2 + s^2 = R_\ell ^2 + (1-2R_\ell ^2) s^2 = \frac{\ell +2s^2}{2\ell +2} , \end{aligned}$$

which proves the second equation in the lemma. $\square$

### Ordering the Radii

In this subsection, we show that the radii of the circumspheres of the ideal simplices increase with increasing $\ell$ and *j*:

#### Lemma 2.5

(Ordering of Radii in ${{\mathbb R}}^{2k}$) Let $0< s < 1 / \sqrt{2k}$. Then the ideal simplices satisfy  for $0 \le \ell \le k-2$, and  for $-1 \le j < \ell \le k-1$.

#### Proof

To prove the first inequality, we use Lemma 2.4 to compute the difference between the two squared radii:

7 $$\begin{aligned} {r^{2}_{{\ell +1},{-1}}} (s) - {r^{2}_{{\ell },{\ell }}} (s)&= \frac{\ell +1}{2(\ell +2)} - \frac{\ell +2s^2}{2(\ell +1)} = \frac{1 - 2s^2(\ell +2)}{2 (\ell +2) (\ell +1)} . \end{aligned}$$

Hence, ${r^{2}_{{\ell },{\ell }}} (s) < {r^{2}_{{\ell +1},{-1}}} (s)$ iff $s^2 < 1 / (2\ell +4)$. We need this inequality for $0 \le \ell \le k-2$, so $s^2 < 1 / (2k)$ is sufficient, but this is guaranteed by the assumption.

We prove the second inequality geometrically, without explicit computation of the radii. Fix an ideal simplex, ${\Sigma }_{\ell ,j}$, and let ${S}^{d-1}$ be the $(d-1)$-sphere whose center and radius are the circumcenter and circumradius of ${\Sigma }_{\ell ,j}$. Assume w.l.o.g. that the circles $C_0$ to $C_j$ contain two vertices of ${\Sigma }_{\ell ,j}$ each, and the circles $C_{j+1}$ to $C_\ell$ contain one vertex of ${\Sigma }_{\ell ,j}$ each. For $0 \le i \le k-1$, write $P_i$ for the 2-plane that contains $C_i$ and $x_i$ for the projection of the center of ${S}^{d-1}$ onto $P_i$. Note that ${\Vert {x_i}\Vert }^2$ is the squared distance to the origin, and for $0 \le i \le \ell$ write $r_i^2$ for the squared distance between $x_i$ and the one or two vertices of ${\Sigma }_{\ell ,j}$ in $P_i$. Fixing *i* between 0 and $\ell$, the squared radius of ${S}^{d-1}$ is $r_i^2$ plus the squared distance of the center of ${S}^{d-1}$ from $P_i$, which is the sum of the squared norms other than ${\Vert {x_i}\Vert }^2$. Taking the sum for $0 \le i \le \ell$ and dividing by $\ell +1$, we get

8 $$\begin{aligned} {r^{2}_{{\ell },{j}}} (s)&= \frac{1}{\ell +1} \left[ \sum \nolimits _{i=0}^{\ell } r_i^2 + \ell \cdot \sum \nolimits _{i=0}^{\ell } {\Vert {x_i}\Vert }^2 + (\ell +1) \cdot \sum \nolimits _{i={\ell +1}}^{k-1} {\Vert {x_i}\Vert }^2 \right] . \end{aligned}$$

By construction, ${r^{2}_{{\ell },{j}}} (s)$ is the minimum squared radius of any $(d-1)$-sphere that passes through the vertices of ${\Sigma }_{\ell ,j}$. Hence, also the right-hand side of (8) is a minimum, but since the 2-planes are pairwise orthogonal, we can minimize in each 2-plane independently of the other. For $\ell +1 \le i \le k-1$, this implies ${\Vert {x_i}\Vert }^2 = 0$, so we can drop the last sum in (8). For $j+1 \le i \le \ell$, $x_i$ lies on the line passing through the one vertex in $P_i$ and the origin. This implies that ${S}^{d-1}$ touches $C_i$ at this vertex, and all other points of the circle lie strictly outside ${S}^{d-1}$. For $0 \le i \le j$, $x_i$ lies on the bisector line of the two vertices, which passes through the origin. The contribution to (8) for an index between 0 and *j* is thus strictly larger than for an index between $j+1$ and $\ell$. This finally implies ${r^{2}_{{\ell },{j}}} (s) < {r^{2}_{{\ell },{j+1}}} (s)$ and completes the proof of the second inequality. $\square$

Recall that 2*s* is the edge length of a regular *n*-gon inscribed in a circle of radius $\sqrt{2}/2$. By the definition of $n_k$, the condition $s < 1 / \sqrt{2k}$ in the lemma holds, whenever $n \ge n_k$.

For the counting argument in the next subsection, we need the ordering of the radii as defined by the radius function, but it is now easy to see that they are the same as the circumradii, so Lemma 2.5 applies. Indeed,  if ${\Sigma }_{\ell ,j}$ is a critical simplex of ${\textrm{Rad}}$. To realize that it is, we note that the circumcenter of ${\Sigma }_{\ell ,j}$ lies in its interior because of symmetry. To see that also the second condition for criticality in Definition 2.2 is satisfied, we recall that ${S}^{d-1}$ is the $(d-1)$-sphere whose center and radius are the circumcenter and circumradius of ${\Sigma }_{\ell ,j}$. By the argument in the proof of Lemma 2.5, ${S}^{d-1}$ is empty, and all points of *A* other than the vertices of ${\Sigma }_{\ell ,j}$ lie strictly outside this sphere.

### Counting the Cycles

To compute the Betti numbers, we make essential use of the structure of the Delaunay mosaic of *A*, which consists of as many groups of congruent ideal simplices as there are different values of the radius function. For each $0 \le \ell \le k-1$, we have $\ell +2$ groups of simplices that touch exactly $\ell +1$ of the *k* circles. In addition, we have a single 2*k*-cell, ${\mathrm{conv\,}{A}}$, with radius , which gives $$1 + 2 + \ldots + (k+1) = \left( {\begin{array}{c}k+2\\ 2\end{array}}\right) $$ groups. We write  for the Alpha complex that consists of all simplices with circumradii at most . We prove Theorem 2.1 in two steps, first the relations (1) for $0 \le p \le k-1$ and second the relations (2) for $k \le p \le 2k-2$. The case $p = 2k-1$ will be settled later, in Section 4.6. To begin, we study the Alpha complexes whose simplices touch at most $\ell +1$ of the *k* circles.

#### Lemma 2.6

(Constant Homology in ${{\mathbb R}}^{2k}$) Let *k* be a constant, $A = A_{2k} (n) \subseteq {{\mathbb R}}^{2k}$, and $0 \le \ell \le k-1$. Then ${\beta }_{p}{({\mathcal {A}_{{\ell },{\ell }}})} = O(1)$ for every $0 \le p \le 2k-1$.

#### Proof

Fix $\ell$ and a subset of $\ell +1$ circles. The full subcomplex of ${\mathcal {A}_{{\ell },{\ell }}}$ defined by the points of *A* on these $\ell +1$ circles consists of all cells in ${\textrm{Del}{({A})}}$ whose vertices lie on these and not any of the other circles. Its homotopy type is that of the join of $\ell +1$ circles or, equivalently, that of the $(2\ell +1)$-sphere; see [16, pages 9 and 19]. This sphere has only one non-zero (reduced) Betti number, which is ${\beta }_{2\ell +1}{} = 1$. There are $$\left( {\begin{array}{c}k\\ \ell +1\end{array}}\right) $$ such full subcomplexes. The common intersection of any number of these subcomplexes is a complex of similar type, namely the full subcomplex of ${\textrm{Del}{({A})}}$ defined by the points on the common circles, which has the homotopy type of the $(2i+1)$-sphere, with $i \le \ell$. By repeated application of the Mayer–Vietoris sequence [16, page 149], this implies that the Betti numbers of ${\mathcal {A}_{{\ell },{\ell }}}$ are bounded by a function of *k* and are, thus, independent of *n*. Since we assume that *k* is a constant, we have ${\beta }_{p}{({\mathcal {A}_{{\ell },{\ell }}})} = O(1)$ for every *p*. $\square$

Now we are ready to complete the proof of Theorem 2.1 for $p \le 2k-2$. To establish relation (1), fix *p* between 0 and $k-1$ and consider , which is the Alpha complex consisting of all simplices that touch *p* or fewer circles, together with all simplices that touch $p+1$ circles but each circle in only one point. In other words, ${\mathcal {A}_{{p},{-1}}}$ is ${\mathcal {A}_{{p-1},{p-1}}}$ together with all the $$\left( {\begin{array}{c}k\\ p+1\end{array}}\right) n^{p+1}$$ *p*-simplices that have no short edges. By Lemma 2.6, ${\mathcal {A}_{{p-1},{p-1}}}$ has only a constant number of $(p-1)$-cycles. Hence, only a constant number of the *p*-simplices can give death to $(p-1)$-cycles, while the remaining *p*-simplices give birth to *p*-cycles. This is because every *p*-simplex either gives birth or death, so if it cannot give death to a $(p-1)$-cycle, then it gives birth to a *p*-cycle. Hence, $${\beta }_{p}{({\mathcal {A}_{{p},{-1}}})} = \left( {\begin{array}{c}k\\ p+1\end{array}}\right) n^{p+1} \pm O(1)$$, as claimed. The proof of relation (2) is similar but inductive. The induction hypothesis is

9 $$\begin{aligned} {\beta }_{p}{({\mathcal {A}_{{k-1},{p-k}}})}&= \genfrac(){0.0pt}1{k-1}{p-k+1} \cdot n^k \pm O(1) . \end{aligned}$$

For $p = k-1$, it claims ${\beta }_{k-1}{({\mathcal {A}_{{k-1},{-1}}})} = n^k \pm O(1)$, which is what we just proved. In other words, relation (1) furnishes the base case at $p = k-1$. A single inductive step takes us from ${\mathcal {A}_{{k-1},{p-k}}}$ to ${\mathcal {A}_{{k-1},{p-k+1}}}$; that is: we add all simplices that touch all *k* circles and $p-k+2$ of them in two vertices to ${\mathcal {A}_{{k-1},{p-k}}}$. The number of such simplices is the number of ways we can pick a pair of consecutive vertices from $p-k+2$ circles and a single vertex from the remaining $2k-p-2$ circles. Since there are equally many vertices as there are consecutive pairs, this number is $$\left( {\begin{array}{c}k\\ p-k+2\end{array}}\right) n^k$$. The dimension of these simplices is $(k-1)+(p-k+1)+1 = p+1$. Some of these $(p+1)$-simplices give death to *p*-cycles, while the others give birth to $(p+1)$-cycles in ${\mathcal {A}_{{k-1},{p-k+1}}}$. By the induction hypothesis, there are $$\left( {\begin{array}{c}k-1\\ p-k+1\end{array}}\right) \cdot n^k \pm O(1)$$ *p*-cycles in ${\mathcal {A}_{{k-1},{p-k}}}$, so this is also the number of $(p+1)$-simplices that give death. Since $$\left( {\begin{array}{c}k\\ p-k+2\end{array}}\right) - \left( {\begin{array}{c}k-1\\ p-k+1\end{array}}\right) = \left( {\begin{array}{c}k-1\\ p-k+2\end{array}}\right) $$, this implies

10 $$\begin{aligned} {\beta }_{p}{({\mathcal {A}_{{k-1},{p-k+1}}})}&= \genfrac(){0.0pt}1{k-1}{p-k+2} \cdot n^k \pm O(1) , \end{aligned}$$

as required to finish the inductive argument.

## Three Dimensions

In this section, we answer the maximum Betti number question for Čech complexes in the smallest odd dimension in which it is non-trivial:

### Theorem 3.1

(Maximum Betti Numbers in ${{\mathbb R}}^3$) For every $n \ge 2$, there exist $N = 2n+2$ points in ${{\mathbb R}}^3$ and two radii such that the Čech complex for the first radius has first Betti number ${\beta }_{1}{} = (n+1)^2 - 1$ and for the second radius has second Betti number ${\beta }_{2}{} = n^2$.

The proof consists of four steps: the construction of the set in Section 3.1, the analysis of the circumradii in Section 3.2, the argument that all simplices in the Delaunay mosaic are critical in Section 3.3, and the final counting of the tunnels and voids in Section 3.4.

### Construction

**Fig. 1:**
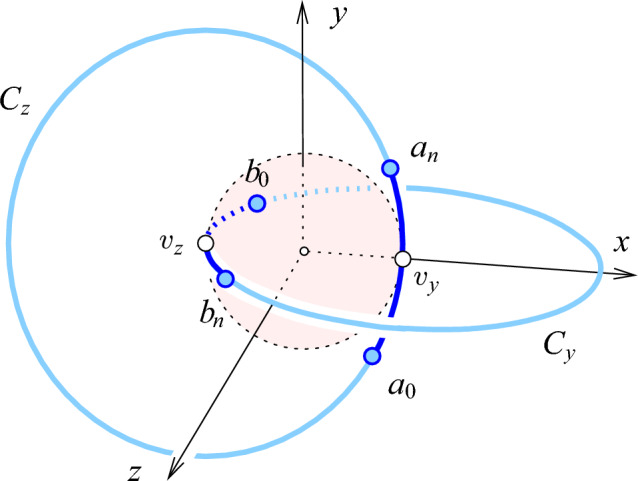
Two linked unit circles in orthogonal coordinate planes of ${{\mathbb R}}^3$, each touching the shaded sphere centered at the origin and each passing through the center of the other circle. There are $n+1$ points on each circle, on both sides and near the center of the other circle

Given *n* and $0< \Delta < 1$, we construct the point set, $A = A_3 (n,\Delta )$, using two linked circles in ${{\mathbb R}}^3$: ${{C}_{z}}$ with center $v_z = (-\frac{1}{2}, 0, 0)$ in the *xy*-plane defined by $(-\frac{1}{2} + \cos \varphi , \sin \varphi , 0)$ for $0 \le \varphi < 2 \pi$, and ${{C}_{y}}$ with center $v_y = (\frac{1}{2}, 0, 0)$ in the *xz*-plane defined by $(\frac{1}{2} - \cos \psi , 0, \sin \psi )$ for $0 \le \psi < 2 \pi$; see Figure 1. On each circle, we choose $n+1$ points close to the center of the other circle. To be specific, take the points $(0,-\Delta ,0)$ and $(0,\Delta ,0)$, and project them to ${{C}_{z}}$ along the *x*-axis. The resulting points are denoted by $a_0 = (-\frac{1}{2}+\sqrt{1-\Delta ^2},-\Delta ,0)$ and $a_n = (-\frac{1}{2}+\sqrt{1-\Delta ^2},\Delta ,0)$. Divide the arc between them into *n* equal pieces by placing the points $a_1, a_2, \ldots , a_{n-1}$ in this sequence from $a_0$ to $a_n$. Symmetrically, project the points $(0,0,-\Delta )$ and $(0,0,\Delta )$ to $b_0 = (\frac{1}{2}-\sqrt{1-\Delta ^2},0,-\Delta )$ and $b_n = (\frac{1}{2}-\sqrt{1-\Delta ^2},0,\Delta )$ lying on ${{C}_{y}}$, and place points $b_1, b_2, \ldots , b_{n-1}$ in this sequence between them, thus dividing the arc from $b_0$ to $b_n$ into *n* equal pieces. Let ${\varepsilon }= {\varepsilon }(n,\Delta )$ be the half-length of the (straight) edge connecting two consecutive points of either sequence. Clearly, ${\varepsilon }$ is a function of *n* and $\Delta$, and it is easy to see that

11 $$\begin{aligned} \Delta / n< {\varepsilon }< \tfrac{\pi }{2} \Delta / n \text{ and } {\varepsilon }{\mathop {\longrightarrow }\limits ^{\Delta \rightarrow 0}} \Delta /n . \end{aligned}$$

A sphere that does not contain a circle intersects it in at most two points. It follows that the sphere that passes through four points of *A* is empty if and only if two of the four points are consecutive on one circle and the other two are consecutive on the other. This determines the Delaunay mosaic: its $N = 2n+2$ vertices are the points $a_i$ and $b_j$, its $2n + (n+1)^2$ edges are of the forms $a_i a_{i+1}$, $b_j b_{j+1}$, and $a_i b_j$, its $2n(n+1)$ triangles are of the forms $a_i a_{i+1} b_j$ and $a_i b_j b_{j+1}$, and its $n^2$ tetrahedra of the form $a_i a_{i+1} b_j b_{j+1}$. Keeping with the terminology introduced in Section 2, we call the edges $a_ib_j$ *long* and the edges $a_ia_{i+1}$ and $b_j b_{j+1}$ *short*. Hence, every triangle in the Delaunay mosaic has one short and two long edges, and every tetrahedron has two short and four long edges.

### Divergence from the Ideal

The simplices in ${\textrm{Del}{({A})}}$ are not quite ideal, in the sense of Section 2. We, therefore, need upper and lower bounds on their sizes, as quantified by their circumradii. We will make repeated use of the following two inequalities, which both hold for $x > -1$:

12 $$\begin{aligned} \sqrt{1+x}&\le 1 + \tfrac{x}{2} , \end{aligned}$$

13 $$\begin{aligned} \sqrt{1+x}&\ge 1 + \tfrac{x}{2+x} . \end{aligned}$$

To begin, we rewrite the relations for the ideal triangle and tetrahedron. Setting $x = s^2 / (1-s^2)$ and $y = 2s^2$, we get $4 r^2(s) = 1+x$ from (3) and $4 R^2(s) = 1 + y$ from (4). Assuming *n* is sufficiently large so that $2 - 2s^2 > 1.9$ and, therefore, $1 + s^2 < 1.1$, we use (12) and (13) to get lower and upper bounds for the two radii:

14 $$\begin{aligned} 1 + \frac{1}{2} s^2< 1 + \frac{s^2 / (1-s^2)}{2 + s^2 / (1-s^2)} \le \,2r(s)\,&\le 1 + \frac{s^2}{2-2s^2} < 1 + \frac{10}{19} s^2 , \end{aligned}$$

15 $$\begin{aligned} 1 + \frac{10}{11} s^2 \le 1 + \frac{s^2}{1+s^2} \le 2R(s)&\le 1+s^2 , \end{aligned}$$

where we apply (12) and (13) to get the inequalities on the right-hand and left-hand sides, respectively. These inequalities are instrumental in deriving bounds in ${{\mathbb R}}^3$:

#### Lemma 3.2

(Bounds for Long Edges in ${{\mathbb R}}^3$) Let $0< \Delta < 1$ and $A = A_3 (n, \Delta ) \subseteq {{\mathbb R}}^3$.

Then the half-length of any long edge, $E \in {\textrm{Del}{({A})}}$, satisfies $\frac{1}{2} \le R_E \le \frac{1}{2} (1 + \Delta ^4)$.

#### Proof

To verify the lower bound, let $a \in {{C}_{z}}$ and consider the sphere with unit radius centered at *a*. This sphere intersects the *xz*-plane in a circle of radius at most 1, whose center lies on the *x*-axis. The circle passes through $v_z \in {{C}_{y}}$, which implies that the rest of ${{C}_{y}}$ lies on or outside the circle and, therefore, on or outside the sphere centered at *a*. Hence, ${\Vert {a}-{b}\Vert } \ge 1$ for all $b \in {{C}_{y}}$, which implies the required lower bound.

To establish the upper bound, observe that the distance between *a* and *b* is maximized if the two points are chosen as far as possible from the *x*-axis, so $4 R_E^2 \le {\Vert {a_0}-{b_0}\Vert }^2$. By construction, $a_0 = (-\frac{1}{2}+\sqrt{1-\Delta ^2}, - \Delta , 0)$ and $b_0 = ( \frac{1}{2}-\sqrt{1-\Delta ^2}, 0, -\Delta )$. Hence,

16 $$\begin{aligned} 4 R_E^2&\le {\Vert {(-1+2\sqrt{1-\Delta ^2}, -\Delta , \Delta )}\Vert }^2 = 5 - 2\Delta ^2 - 4\sqrt{1-\Delta ^2} \end{aligned}$$

17 $$\begin{aligned}&\le 5 - 2\Delta ^2 - 4\left( 1 - \frac{\Delta ^2}{2-\Delta ^2} \right) = 1 + \frac{2\Delta ^4}{2-\Delta ^2} \end{aligned}$$

18 $$\begin{aligned}&\le 1 + 2 \Delta ^4 , \end{aligned}$$

where we used (13) to get (17) from (16), and $\Delta ^2 < 1$ to obtain the final bound. Applying (12), wet get $2R_E \le 1 + \Delta ^4$, as required. $\square$

Next, we estimate the circumradii of the triangles in ${\textrm{Del}{({A})}}$. To avoid the computation of a constant, we use the big-Oh notation for $\Delta$, in which we assume that *n* is fixed.

#### Lemma 3.3

(Bounds for Triangles in ${{\mathbb R}}^3$) Let $0< \Delta < \sqrt{2}/n$, $A = A_3 (n,\Delta ) \subseteq {{\mathbb R}}^3$, and ${\varepsilon }= {\varepsilon }(n,\Delta )$.

Then the circumradius of any triangle, *F*, satisfies $\frac{1}{2} + \frac{1}{4} {\varepsilon }^2 \le R_F \le \frac{1}{2} + \frac{1}{4} {\varepsilon }^2 +O(\Delta ^4).$

#### Proof

To see the lower bound, recall that the short edge of *F* has length $2 {\varepsilon }$ and the two long edges have lengths at least 1. A circle of radius $r({\varepsilon })$ that passes through the endpoints of the short edge has only one point at distance at least 1 from both endpoints, and it has distance 1 from both. For any radius smaller than $r({\varepsilon })$, there is no such point, which implies that the circumradius of *F* satisfies $R_F \ge r({\varepsilon }) \ge \frac{1}{2} + \frac{1}{4} {\varepsilon }^2$, where the second inequality follows from (14).

To prove the upper bound, we draw *F* in the plane, assuming its circumcircle is the circle with radius $R_F$ centered at the origin. Let *a*, *b*, *c* be the vertices of *F*, where *a* and *c* are the endpoints of the short edge. We have $0 \in F$, since otherwise one of the angles at *a* and *c* is obtuse, in which case the squared lengths of the two long edges differ by at least $4 {\varepsilon }^2$. By assumption, $\sqrt{2} \Delta ^2 < 2\Delta /n \le 2 {\varepsilon }$, in which we get the second inequality from (11). But this implies that the difference between the squared lengths of the two long edges is larger than $2 \Delta ^4$, which contradicts Lemma 3.2. Hence, *b* lies between the antipodes of the other two vertices, $a' = -a$ and $c' = -c$. By construction, ${\Vert {a'}-{c'}\Vert } = 2{\varepsilon }$. Assuming ${\Vert {b}-{a'}\Vert } \le {\Vert {b}-{c'}\Vert }$, this implies

19 $$\begin{aligned} {\Vert {b}-{a'}\Vert }&\le 2 R_F \arcsin \tfrac{{\varepsilon }}{2 R_F} \le \arcsin {\varepsilon }= {\varepsilon }+ O({\varepsilon }^3). \end{aligned}$$

Here, the second inequality follows from $2 R_F \ge 1$, using the convexity of the arcsin function, and the final expression using the Taylor expansion $\arcsin x = x + \frac{1}{6} x^3 + \frac{3}{40} x^5 + \ldots$. Now consider the triangle with vertices $a, a', b$. By the Pythagorean theorem,

20 $$\begin{aligned} 4 R_F^2&= {\Vert {b}-{a}\Vert }^2 + {\Vert {b}-{a'}\Vert }^2 < 1 + 2 \Delta ^4 + \Delta ^8 + {\varepsilon }^2 + O({\varepsilon }^4) = 1 + {\varepsilon }^2 + O(\Delta ^4) , \end{aligned}$$

where we used Lemma 3.2 and (19) to bound ${\Vert {b}-{a}\Vert }^2$ and ${\Vert {b}-{a'}\Vert }^2$, respectively. We get the final expression using ${\varepsilon }< \Delta$. Applying (12), we obtain $2 R_F \le 1 + \frac{1}{2} {\varepsilon }^2 + O(\Delta ^4)$, as claimed. $\square$

Similar to the case of triangles, it is not difficult to establish that the circumradius of any tetrahedron in the Delaunay mosaic is at least the circumradius of the ideal tetrahedron.

#### Lemma 3.4

(Lower Bound for Tetrahedra in ${{\mathbb R}}^3$) Let $0< \Delta < 1$, $A = A_3 (n,\Delta ) \subseteq {{\mathbb R}}^3$, and ${\varepsilon }= {\varepsilon }(n,\Delta )$.

Then the circumradius of any tetrahedron $T \in {\textrm{Del}{({A})}}$ satisfies $R_T \ge \frac{1}{2} + \frac{5}{11} {\varepsilon }^2$.

#### Proof

By construction, *T* has two disjoint short edges, both of length $2 {\varepsilon }$. Consider a sphere of radius $R({\varepsilon })$ that passes through the endpoints of one of the two short edges. The set of points on this sphere that are at distance at least 1 from both endpoints is the intersection of two spherical caps whose centers are antipodal to the endpoints. We call this intersection a *spherical bi-gon*. Since the two caps have the same size, the two corners of the bi-gon are further apart than any other two points of the bi-gon. By choice of the radius, $R({\varepsilon })$, the edge connecting the two corners has length $2 {\varepsilon }$. Hence, these corners are the only possible choice for the remaining two vertices of *T*, and for a radius smaller than $R({\varepsilon })$, there is no choice. It follows that the circumradius of *T* is at least $R({\varepsilon })$, and we get the claimed lower bound from (15). $\square$

### All Simplices are Critical

Since no empty sphere passes through more than four points of *A*, the Delaunay mosaic of *A* is simplicial, and the radius function is a generalized discrete Morse function [4]. We will argue shortly that all simplices are critical; see Definition 2.2. The point set depends on two parameters, *n* and $\Delta$, and we consider *n* fixed while we can make $\Delta$ as small as we like.

#### Lemma 3.5

(All Critical in ${{\mathbb R}}^3$) Let $n \ge 2$, $\Delta > 0$ sufficiently small, and $A = A_3 (n,\Delta ) \subseteq {{\mathbb R}}^3$. Then every simplex of the Delaunay mosaic of *A* is critical.

#### Proof

It is clear that the vertices and the short edges are critical, but the other simplices in ${\textrm{Del}{({A})}}$ require an argument. We begin with the long edges. Fix *i* and *j*, and write ${S}^{2} (i; j)$ for the smallest sphere that passes through $a_i$ and $b_j$. Its center is the midpoint of the long edge and, by (18), its squared diameter is between 1 and $1 + 2 \Delta ^4$. The distance between $a_i$ and any $a_\ell$, $\ell \ne i$, is at least $2 {\varepsilon }$. Assuming $a_\ell$ is on or inside ${S}^{2} (i;j)$, we thus have ${\Vert {a_\ell }-{b_j}\Vert }^2 \le 1 + 2 \Delta ^4 - 4 {\varepsilon }^2$, which, for sufficiently small $\Delta > 0$, is less than 1. This contradicts the lower bound in Lemma 3.2, so $a_\ell$ lies outside ${S}^{2} (i;j)$. By a symmetric argument, all $b_\ell$, $\ell \ne j$, lie outside ${S}^{2} (i;j)$. Hence, ${S}^{2} (i;j)$ is strictly empty, for all $0 \le i, j \le n$, which implies that all edges of ${\textrm{Del}{({A})}}$ are critical edges of the radius function.

**Fig. 2:**
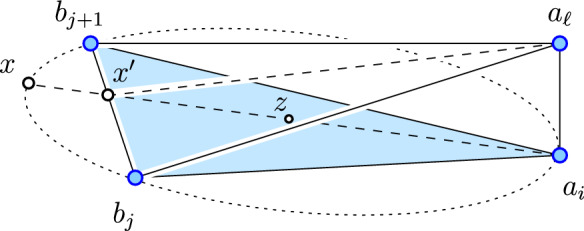
Two acute triangles sharing the edge that connects $b_j$ with $b_{j+1}$ in ${\textrm{Del}{({A})}}$. By shrinking $\Delta > 0$, the angle at $x'$ can be made arbitrarily close to straight and certainly larger than $\frac{\pi }{2}$

The fact that all edges of ${\textrm{Del}{({A})}}$ are critical implies that all triangles are acute. Indeed, if $a_i b_j b_{j+1}$ is not acute, then the midpoint of one long edge is at least as close to the third vertex as to the endpoints of the edge. Write ${S}^{2} (i; j, j+1)$ for the circumsphere of the triangle and *z* for its center. Since $a_i b_j b_{j+1}$ is acute, *z* lies in its interior. As illustrated in Figure 2, the line that passes through $a_i$ and *z* crosses the opposite edge at $x'$ and exits the sphere at *x*. Let $a_\ell$ be another point, with $\ell \ne i$, and assume it lies on or outside ${S}^{2} (i; j, j+1)$. The angle between the segments that connect $a_\ell$ to $a_i$ and *x* is therefore at least $\frac{\pi }{2}$, which implies

21 $$\begin{aligned} {\Vert {x}-{a_i}\Vert }^2&\ge {\Vert {x}-{a_\ell }\Vert }^2 + {\Vert {a_i}-{a_\ell }\Vert }^2 \ge 1 - {\varepsilon }^2 + 4 {\varepsilon }^2 = 1 + 3 {\varepsilon }^2 , \end{aligned}$$

because the angle enclosed by the segments connecting $x'$ to $a_\ell$ and *x* is larger than $\frac{\pi }{2}$, so ${\Vert {x}-{a_\ell }\Vert }^2$ is larger than the squared height of the triangle $a_\ell b_j b_{j+1}$, which is at least $1 - {\varepsilon }^2$, and because ${\Vert {a_i}-{a_\ell }\Vert }^2 \ge 4 {\varepsilon }^2$. But (21) contradicts ${\Vert {x}-{a_i}\Vert }^2 \le 1 + {\varepsilon }^2 + O(\Delta ^4)$, which follows from the upper bound on the radius of the triangle in Lemma 3.3. Hence, all triangles in ${\textrm{Del}{({A})}}$ are critical, as claimed.

Since all triangles are critical, all tetrahedra of ${\textrm{Del}{({A})}}$ must also be critical. One can argue in two ways. Combinatorially: the radius function pairs non-critical tetrahedra with non-critical triangles, but there are no such triangles. Geometrically: since every triangle has a non-empty intersection with its dual Voronoi edge, every tetrahedron must contain its dual Voronoi vertex. $\square$

### Counting the Tunnels and Voids

Before counting the tunnels and voids, we recall that ${\textrm{Rad}}:{\textrm{Del}{({A})}} \rightarrow {{\mathbb R}}$ maps each simplex to the radius of its smallest empty sphere that passes through its vertices. By Lemma 3.5, all simplices of ${\textrm{Del}{({A})}}$ are critical, so ${\textrm{Rad}}(E)$ is equal to the circumradius of *E*, for every edge $E \in {\textrm{Del}{({A})}}$, and similarly for every triangle and every tetrahedron.

#### Corollary 3.6

(Ordering of Radii in ${{\mathbb R}}^3$) Let $\Delta > 0$ be sufficiently small, let $A = A_3 (n,\Delta ) \subseteq {{\mathbb R}}^3$, and let ${\textrm{Rad}}:{\textrm{Del}{({A})}} \rightarrow {{\mathbb R}}$ be the radius function. Then ${\textrm{Rad}}(E)< {\textrm{Rad}}(F) < {\textrm{Rad}}(T)$ for every edge *E*, triangle *F*, and tetrahedron *T* in ${\textrm{Del}{({A})}}$.

#### Proof

Using Lemma 3.2 for the edges, Lemma 3.3 for the triangles, and Lemma 3.4 for the tetrahedra in the Delaunay mosaic of *A*, we get

22 $$\begin{aligned} {\textrm{Rad}}(E) = R_E&< \tfrac{1}{2} + O(\Delta ^4), \end{aligned}$$

23 $$\begin{aligned} \tfrac{1}{2} + \tfrac{1}{4} {\varepsilon }^2 \le {\textrm{Rad}}(F) = R_F&< \tfrac{1}{2} + \tfrac{1}{4} {\varepsilon }^2 + O(\Delta ^4), \end{aligned}$$

24 $$\begin{aligned} \tfrac{1}{2} + \tfrac{5}{11} {\varepsilon }^2 \le {\textrm{Rad}}(T) = R_T&, \end{aligned}$$

so for sufficiently small $\Delta > 0$, the edges precede the triangles, and the triangles precede the tetrahedra in the filtration of the simplices. $\square$

For the final counting, choose $\rho _1$ to be any number strictly between the maximum radius of any edge and the minimum radius of any triangle. The existence of such a number is guaranteed by Corollary 3.6. The corresponding Čech complex is the 1-skeleton of the Delaunay mosaic. It is connected, with $N = 2n+2$ vertices and $2n + (n+1)^2$ edges. The number of independent cycles is the difference plus 1, which implies , as claimed. Similarly, choose $\rho _2$ between the maximum radius of any triangle and the minimum radius of any tetrahedron, which is again possible, by Corollary 3.6. The corresponding Čech complex is the 2-skeleton of the Delaunay mosaic. The number of independent 2-cycles is the number of missing tetrahedra. This implies , as claimed.

## Odd Dimensions

In this section, we generalize the 3-dimensional results to odd dimensions and, in Section 4.6, we prove the outstanding case, $p = 2k-1$ and $d = 2k$, in even dimensions.

### Theorem 4.1

(Maximum Betti Numbers in ${{\mathbb R}}^{2k+1}$) For every $d = 2k+1 \ge 1$, $n \ge 2$, and sufficiently small $\Delta > 0$, there are a set $A = A_d(n,\Delta ) \subseteq {{\mathbb R}}^{2k+1}$ of $N = (k+1)(n+1)$ points and radii $\rho _0< \rho _1< \ldots < \rho _{2k}$ such that2526

The steps in the proof are the same as in Sections 2 and 3: construction of the points, analysis of the circumradii, argument that all simplices are critical, and final counting of the cycles. In contrast to the earlier sections, the analytic part of the proof is inductive and distinguishes between erecting a pyramid or a bi-pyramid on top of a lower-dimensional simplex.

### Construction

**Fig. 3:**
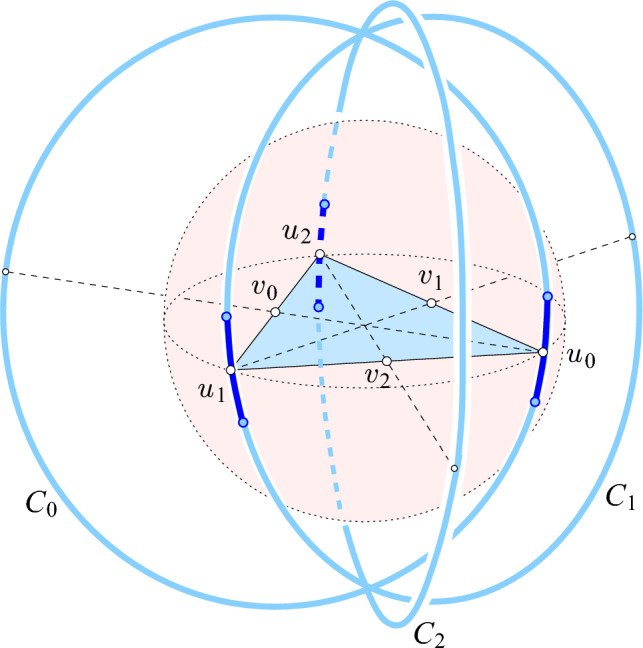
The projection of the 5-dimensional construction to ${{\mathbb R}}^3$, in which $x_3, x_4, x_5$ are all mapped to the same, vertical coordinate direction. The circles ${{C}_{0}}, {{C}_{1}}, {{C}_{2}}$ touch the shaded sphere in the vertices of the triangle. In ${{\mathbb R}}^5$, the three circles belong to mutually orthogonal 2-planes, so the two common points of the three circles in the drawing are an artifact of the particular projection

Equip ${{\mathbb R}}^d$ with Cartesian coordinates, $x_1, x_2, \ldots , x_d$, and consider a regular *k*-simplex, denoted by , in the *k*-plane spanned by $x_1, x_2, \ldots , x_k$. It is not important where  is located inside the coordinate *k*-plane, but we assume for convenience that its barycenter is the origin of the coordinate system. It is, however, important that all edges of $\Sigma$ have unit length. We will repeatedly need the squared circumradius, height, and in-radius of $\Sigma$, for which we state simple formulas and straightforward consequences for later convenience:

27 $$\begin{aligned} R_k^2&= \tfrac{k}{2(k+1)}; \hspace{0.58in} D_k^2 = \tfrac{1}{2 k (k+1)} ; \hspace{0.73in} H_k^2 = \tfrac{k+1}{2k} ; \end{aligned}$$

28 $$\begin{aligned} (k + 1) R_k&= k H_k; ~~~ (k + 1) R_{k-1}^2 = (k - 1) H_k^2; ~~~ (k + 1) D_k = H_k, \end{aligned}$$

in which we get the second equation in (27) from $D_k^2 = R_k^2 - R_{k-1}^2$. Observe that the angle, $\alpha$, between an edge and a height of  that meet at a shared vertex satisfies $\cos \alpha = H_k$. Let $u_0, u_1, \ldots , u_k$ be the vertices of , and let $v_\ell$ be the barycenter of the $(k-1)$-face opposite to $u_\ell$. For each $0 \le \ell \le k$, consider the 2-plane spanned by $u_\ell - v_\ell$ and the $x_{k+\ell +1}$-axis, and let ${{C}_{\ell }}$ be the circle in this 2-plane, centered at $v_\ell$, that passes through $u_\ell$; see Figure 3. Its radius is the height of the *k*-simplex: ${\gamma }= H_k$. Given a global choice of the parameter, $0< \Delta < H_k$, we cut ${{C}_{\ell }}$ at $x_{k+\ell +1} = \pm \Delta$ into four arcs and place $n+1$ point at equal angles along the arc that passes through $u_\ell$. Repeating this step for each $\ell$, we get a set of $N = (k+1)(n+1)$ points, denoted $A = A_{2k+1} (n,\Delta )$.

A $(d-1)$-sphere that contains none of the circles ${{C}_{\ell }}$ intersects the $k+1$ circles in at most two points each. It follows that a sphere that passes through $2k+2$ points of $A_d$ is empty if and only if it passes through two consecutive points on each of the $k+1$ circles. This determines the Delaunay mosaic, which consists of $n^{k+1}$ *d*-simplices together with all their faces. It follows that the number of *p*-simplices in ${\textrm{Del}{({A})}}$ is at most some constant times $n^m$, in which $m = \min \{ p+1, k+1 \}$ and the constant depends on $d = 2k+1$. Building on the notation introduced in Section 2, we describe each simplex, $S \in {\textrm{Del}{({A})}}$, with two integers: $\ell = {{\ell }{({S})}}$ is one less than the number of circles ${{C}_{\ell }}$ that each contain one or two vertices of *S*, and $j = {{j}{({S})}}$ is one less than the number of circles that each contain two vertices of *S*. Hence, *S* has dimension $p = \ell + 1 + j$, and $j + 1$ of its edges are short. For each $0 \le p \le k$, there are $$\left( {\begin{array}{c}k+1\\ p+1\end{array}}\right) (n+1)^{p+1}$$ *p*-simplices that touch $\ell +1 = p+1$ circles and thus have $j+1 = 0$ short edges. As suggested by a comparison with relation (25) in Theorem 4.1, these *p*-simplices will be found responsible for the *p*-cycles counted by the *p*-th Betti number.

### Distance from the Ideal

The simplices we work with in odd dimensions are almost but not quite ideal. We quantify the difference by projecting a vertex orthogonally onto the affine hull of a face and measuring the distance between the projected vertex and the circumcenter of the face. We will see that this distance is small provided the face is *far* from the vertex, by which we mean that all edges connecting the vertex to the face are long. We prove this by first establishing bound on the lengths of long edges.

#### Lemma 4.2

(Length of Long Edges in ${{\mathbb R}}^{2k+1}$) Let $d = 2k+1$, $0< \Delta < 1$, and $A = A_d (n,\Delta ) \subseteq {{\mathbb R}}^d$. Then the squared length of any long edge satisfies $1 \le 4 R_E^2 \le 1 + 2 \Delta ^4$.

#### Proof

The length of *E* is maximized if its endpoints, *a* and *b*, are as far as possible from the affine hull of . We therefore assume that both points have distance $\Delta$ from this plane. Suppose $a \in {{C}_{0}}$ and $b \in {{C}_{1}}$, and write $a'$ and $b'$ for their projections onto . Recall that $u_0$ is the point shared by  and ${{C}_{0}}$, and note that ${\Vert {a'}-{u_0}\Vert } = \xi = {\gamma }- \sqrt{{\gamma }^2 - \Delta ^2}$, in which ${\gamma }$ is the radius of $C_0$. Similarly, ${\Vert {b'}-{u_1}\Vert } = \xi$. Let $\alpha$ be the angle enclosed by an edge of  and a height of  that shares a vertex with the edge. Set $\eta = \xi \cos \alpha$ and note that ${\Vert {a'}-{b'}\Vert } = 1-2\eta$. By construction of  as a regular simplex with unit length edges, we have $\cos \alpha = {\gamma }$, so

29 $$\begin{aligned} {\Vert {a}-{b}\Vert }^2&= (1-2\eta )^2 + \Delta ^2 + \Delta ^2 = \left( 1 - 2 {\gamma }^2 + 2 {\gamma }\sqrt{{\gamma }^2 - \Delta ^2} \right) ^2 + 2 \Delta ^2 \end{aligned}$$

30 $$\begin{aligned}&= \left( 1-2{\gamma }^2 \right) ^2 + 4{\gamma }^2 \left( {\gamma }^2 - \Delta ^2 \right) + \left( 2-4{\gamma }^2 \right) 2 {\gamma }\sqrt{{\gamma }^2 - \Delta ^2} + 2 \Delta ^2 \end{aligned}$$

31 $$\begin{aligned}&= \left( 1-4{\gamma }^2+8{\gamma }^4 \right) - \left( 4{\gamma }^2 - 2 \right) \left[ \Delta ^2 + 2{\gamma }\sqrt{{\gamma }^2-\Delta ^2} \right] . \end{aligned}$$

The squared radius of the circles is ${\gamma }^2 = (k+1)/(2k) > \frac{1}{2}$, which implies $4{\gamma }^2 - 2 > 0$. Hence, we can bound ${\Vert {a}-{b}\Vert }^2$ from below using (12) to get $\sqrt{{\gamma }^2-\Delta ^2} \le {\gamma }\left[ 1 - \Delta ^2/(2 {\gamma }^2) \right]$. Plugging this inequality into (31) and applying a sequence of elementary algebraic manipulations gives ${\Vert {a}-{b}\Vert }^2 \ge 1$, as claimed. To prove the upper bound, we use (13) to get $\sqrt{{\gamma }^2-\Delta ^2} \ge {\gamma }\left[ 1 - \Delta ^2/(2{\gamma }^2-\Delta ^2) \right]$. Plugging this inequality into (31) gives

32 $$\begin{aligned} {\Vert {a}-{b}\Vert }^2&\le \left( 1-4{\gamma }^2+8{\gamma }^4 \right) - \left( 4{\gamma }^2-2 \right) \left[ \Delta ^2+2{\gamma }^2- \frac{2 {\gamma }^2 \Delta ^2}{2 {\gamma }^2 - \Delta ^2} \right] \end{aligned}$$

33 $$\begin{aligned}&= 1 + \left( 4 {\gamma }^2 - 2 \right) \frac{\Delta ^4}{2 {\gamma }^2 - \Delta ^2} \le 1 + 2 \Delta ^4 , \end{aligned}$$

where we use $\Delta < 1$ to get the final inequality. $\square$

Applying (12) to the bounds in Lemma 4.2, we get $1 \le 2 R_E \le 1 + \Delta ^4$. Since the length of every short edge is fixed to $2 {\varepsilon }$, and the length of every long edge is tightly controlled, all simplices are almost ideal. The next lemma quantifies this notion.

#### Lemma 4.3

(Distance from Ideal in ${{\mathbb R}}^{2k+1}$) Let $d = 2k+1$, $\Delta > 0$ sufficiently small, $A = A_d (n,\Delta ) \subseteq {{\mathbb R}}^d$, *S* a simplex in ${\textrm{Del}{({A})}}$, *u* a vertex of *S*, and $Q \subseteq S$ a far face of *u*. Then the distance between the orthogonal projection of *u* onto ${\mathrm{aff\,}{Q}}$ and the circumcenter of *Q* is at most $O(\Delta ^3)$.

#### Proof

We begin with a triangle, *S*, with vertices *u*, *v*, *w*, such that the edges connecting *u* to *v* and *w* are both long. The edge connecting *v* to *w* may be long or short. Let $\delta$ be the distance of *u* from the bisector of *v* and *w*, which is maximized if ${\Vert {v}-{w}\Vert }$ is as small as possible while the length difference between the edges connecting *u* to *v* and *w* is as large as possible. Assuming therefore that these two edges have squared lengths 1 and $1 + 2 \Delta ^4$, Pythagoras’ theorem implies $(1+2\Delta ^4) - ({\varepsilon }+\delta )^2 = 1 - ({\varepsilon }-\delta )^2$. Canceling 1, ${\varepsilon }^2$, and $\delta ^2$ on both sides, we get $\Delta ^4 = 2 {\varepsilon }\delta$. Since $n {\varepsilon }\ge \Delta$, this implies $\delta = \Delta ^4 / (2 {\varepsilon }) \le n \Delta ^3 / 2$.

**Fig. 4:**
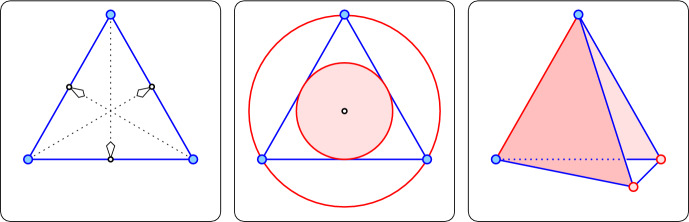
The ingredients for the analysis of the simplices. *Left:* each vertex of the equilateral triangle projects orthogonally to the midpoint of the opposite edge. *Middle:* the largest disk inside the equilateral triangle and concentric with the circumcircle is bounded by the inscribed circle. *Right:* the tetrahedron with one short edge is a bi-pyramid with two apices and one base edge

In other words, the distance between the projection of the vertex and the midpoint of the far edge is $\delta \le n \Delta ^3 /2$; see the left panel in Figure 4. As mentioned earlier, $\Delta$ is independent of *n*, so we write $n \Delta ^3 / 2 = O(\Delta ^3)$, which settles the claim for the triangles in ${\textrm{Del}{({A})}}$.

To generalize beyond triangles, suppose first that the far face of *u* is *i*-dimensional and has no short edges. For each long edge, we construct the slab of points between two parallel hyperplanes, each parallel to and at distance $n \Delta ^3 / 2$ from the normal hyperplane that crosses the edge at its midpoint. As shown above, this slab contains *u*. The common intersection of the slabs of all edges of the face contains *u*, and the further intersection with the affine hull of the face contains the orthogonal projection of *u* onto the face. In the ideal case, this is a centrally symmetric polytope of dimension *i* with $(i+1) i$ facets of dimension $i-1$. The angle between any two adjacent facets is $120^\circ$. For sufficiently small $\Delta > 0$, this angle is only negligibly larger than $120^\circ$, so the polytope is contained in a ball of radius at most some constant times $O(\Delta ^3)$ centered at the circumcenter of the face. By construction, *u* belongs to this ball, which implies the claimed bound for simplices without long edges. Any short edges are almost orthogonal to each other and to the long edges of the face. Each such edge defines a slab, and we can repeat the argument while adding these slabs into the mix. $\square$

### Inductive Analysis

This section continues the analysis with the goals to prove bounds on the circumradii that are strong enough to separate the Delaunay simplices of different types, and to show that all simplices are critical. We use induction, with two hypotheses: the first about the circumradius and the second about the circumcenter. To formulate the second hypothesis, we let *S* be a simplex, and write $D_S$ for the radius of the largest ball contained in *S* that is concentric with the circumsphere of *S*; see the middle panel in Figure 4. If the circumcenter lies outside *S*, then $D_S$ is zero, but we will see that this never happens. Recall that ${\varepsilon }= {\varepsilon }(n,\Delta )$ is a function of *n* and $\Delta$ that satisfies $\Delta /n \le {\varepsilon }\le \frac{\pi }{2} \Delta /n$. We write $\ell +1$ for the number of the ${{C}_{i}}$ touched by *S*, and $j+1$ for the number of short edges.

**Hypothesis I:** $$R_S^2 = R_\ell ^2 + \frac{j+1}{(\ell +1)^2} {\varepsilon }^2 \pm O({\varepsilon }^3)$$.

**Hypothesis II:** $$D_S^2 = \left\{ \begin{array}{cl} D_\ell ^2 \pm O({\varepsilon }^2) &  \text{ if } j = -1; \\ \frac{1}{(\ell +1)^2} {\varepsilon }^2 \pm O({\varepsilon }^3) &  \text{ if } 0 \le j \le \ell , \end{array} \right. $$

in which the big-Oh notation is used to suppress multiplicative constants, as usual. Since $\Delta$ is independent of *n*, we write $\Delta = O({\varepsilon })$. The base case for the induction ascertains that the two hypotheses hold when *S* is a vertex ($\ell = 0$, $j = -1$), a short edge ($\ell = j = 0$), or a long edge ($\ell = 1$, $j = -1$). We have $R_S^2 = 0$ if *S* is a vertex, $R_S^2 = {\varepsilon }^2$ if *S* is a short edge, and $\frac{1}{4} \le R_S^2 \le \frac{1}{4} + \frac{1}{2} \Delta ^4$ if *S* is a long edge by Lemma 4.2, which verify Hypothesis I in all three cases. Hypothesis II is also clear. Indeed, the edge itself is the largest 1-ball contained in the edge and concentric with the circumsphere, so there is nothing to prove.

We will distinguish between two kinds of inductive steps, one reasoning from $(\ell -1,j)$ to $(\ell ,j)$ and the other from $(\ell ,j-1)$ to $(\ell ,j)$. We need some notions to describe the difference. A *facet* of a simplex is a face whose dimension is 1 less than that of the simplex. We call a vertex *a* of *S* a *twin* if it is the endpoint of a short edge, in which case we write $a''$ for the other endpoint of that edge. If *a* is not a twin, we write $Q = S-a$ for the opposite facet, and call the pair (*a*, *Q*) a *pyramid* with *apex* *a* and *base* *Q*. If *a* is a twin, then there are two pyramids, (*a*, *P*) and $(a'',P)$ with $P=S-a-a''$, and we call this the *bi-pyramid case*; see the right panel in Figure 4.

#### Inductive Step (Pyramid Case)

The inductive step consists of two lemmas. The first justifies the inductive step from $(\ell -1,j)$ to $(\ell ,j)$. It handles the transition from the base of a pyramid to the pyramid. Letting *S* be a simplex, $z_S$ its circumcenter, and (*a*, *Q*) be a pyramid of *S*, we write $H_{Q,S}$ and $D_{Q,S}$ for the distances of *a* and $z_S$ from ${\mathrm{aff\,}{Q}}$, respectively.

##### Lemma 4.4

(Pyramid Step) Let $d = 2k+1$, $\Delta > 0$ sufficiently small, $A = A_d (n,\Delta ) \subseteq {{\mathbb R}}^d$, and ${\varepsilon }= {\varepsilon }(n,\Delta )$. Furthermore, let $S \in {\textrm{Del}{({A})}}$, write $\ell = {{\ell }{({S})}}$ and $j = {{j}{({S})}}$, assume $j < \ell$, and let (*a*, *Q*) be a pyramid of *S*. Assuming *Q* satisfies Hypotheses I and II, we have

34 $$\begin{aligned} H_{Q,S}^2&= H_\ell ^2 - \frac{j+1}{\ell ^2} {\varepsilon }^2 \pm O({\varepsilon }^3) ; \end{aligned}$$

35 $$\begin{aligned} D_{Q,S}^2&= D_\ell ^2 - \frac{(2\ell +1)(j+1)}{\ell ^2(\ell +1)^2} {\varepsilon }^2 \pm O({\varepsilon }^3) ; \end{aligned}$$

36 $$\begin{aligned} R_S^2&= R_\ell ^2 + \frac{j+1}{(\ell +1)^2} {\varepsilon }^2 \pm O({\varepsilon }^3) ; \end{aligned}$$

##### Proof

By construction, ${{\ell }{({Q})}} = \ell -1$ and ${{j}{({Q})}} = j$. Assume first that the projection of *a* onto ${\mathrm{aff\,}{Q}}$ is $z_Q$, the circumcenter of *Q*. In this case, all edges connecting *a* to *Q* have the same length, $2 R_E$. Pythagoras’ theorem implies $H_{Q,S}^2 = 4 R_E^2 - R_Q^2$. Using Lemma 4.2 and Hypothesis I, we get the bounds for the squared height claimed in (34):

37 $$\begin{aligned} 4 R_E^2&= 1 \pm O(\Delta ^4) ; \end{aligned}$$

38 $$\begin{aligned} R_Q^2&= R_{\ell -1}^2 + \frac{j+1}{\ell ^2} {\varepsilon }^2 \pm O({\varepsilon }^3) ; \end{aligned}$$

39 $$\begin{aligned} H_{Q,S}^2&= H_\ell ^2 - \frac{j+1}{\ell ^2} {\varepsilon }^2 \pm O({\varepsilon }^3) , \end{aligned}$$

where (39) follows from (37) and (38), using $1 - R_{\ell -1}^2 = H_\ell ^2$. This proves (34). Since $(H_{Q,S} - D_{Q,S})^2 = R_S^2$ and $R_Q^2 + D_{Q,S}^2 = R_S^2$, we get $H_{Q,S}^2 - 2 D_{Q,S} H_{Q,S} = R_Q^2$. Therefore,

40 $$\begin{aligned} D_{Q,S}&= \frac{H_{Q,S}^2 - R_Q^2}{2 H_{Q,S}} ~~= \frac{1}{2} H_{Q,S} - \frac{1}{2} \frac{R_Q^2}{H_{Q,S}} ; \end{aligned}$$

41 $$\begin{aligned} R_S&= H_{Q,S} - D_{Q,S} = \frac{1}{2} H_{Q,S} + \frac{1}{2} \frac{R_Q^2}{H_{Q,S}} . \end{aligned}$$

Using the formulas for $R_\ell$, $H_\ell$, $D_\ell$ in (27), it is easy to prove the corresponding relations for the regular $\ell$-simplex: $D_\ell = \frac{1}{2} H_\ell - \frac{1}{2} R_{\ell -1}^2 / H_\ell$ and $R_\ell = \frac{1}{2} H_\ell + \frac{1}{2} R_{\ell -1}^2 / H_\ell$. Starting with (39), we use $\sqrt{1-x} = 1 - \frac{x}{2} + \ldots$ and $1 / \sqrt{1-x} = 1 + \frac{x}{2} + \ldots$ to get

42 $$\begin{aligned} H_{Q,S}&= H_\ell - \frac{j+1}{2 \ell ^2 H_\ell } {\varepsilon }^2 \pm O({\varepsilon }^3); \end{aligned}$$

43 $$\begin{aligned} \frac{1}{H_{Q,S}}&= \frac{1}{H_\ell } + \frac{j+1}{2 \ell ^2 H_\ell ^3} {\varepsilon }^2 \pm O({\varepsilon }^3) ; \end{aligned}$$

44 $$\begin{aligned} \frac{R_Q^2}{H_{Q,S}}&= \frac{R_{\ell -1}^2}{H_\ell } + \left[ \frac{j+1}{\ell ^2 H_\ell } + \frac{R_{\ell -1}^2 (j+1)}{2 \ell ^2 H_\ell ^3} \right] {\varepsilon }^2 \pm O({\varepsilon }^3) , \end{aligned}$$

where we multiply the left-hand sides and right-hand sides of (38) and (43) to get (44). We plug (42) and (44) into (40) and (41), while using the relations in (27) and (28):

45 $$\begin{aligned} D_{Q,S}&= \left[ \frac{1}{2} H_\ell - \frac{1}{2} \frac{R_{\ell -1}^2}{H_\ell } \right] - \left[ \frac{j+1}{4 \ell ^2 H_\ell } + \frac{j+1}{2 \ell ^2 H_\ell } + \frac{R_{\ell -1}^2 (j+1)}{4 \ell ^2 H_\ell ^3} \right] {\varepsilon }^2 \pm O({\varepsilon }^3) \nonumber \\&= D_\ell - \frac{(2\ell +1)(j+1)}{2 \ell ^2 (\ell +1)^2 D_\ell } {\varepsilon }^2 \pm O({\varepsilon }^3) ; \end{aligned}$$

46 $$\begin{aligned} R_S&= \left[ \frac{1}{2} H_\ell + \frac{1}{2} \frac{R_{\ell -1}^2}{H_\ell } \right] + \left[ - \frac{j+1}{4 \ell ^2 H_\ell } + \frac{j+1}{2 \ell ^2 H_\ell } + \frac{R_{\ell -1}^2 (j+1)}{4 \ell ^2 H_\ell ^3} \right] {\varepsilon }^2 \pm O({\varepsilon }^3) \nonumber \\&= R_\ell + \frac{j+1}{2 (\ell +1)^2 R_\ell } {\varepsilon }^2 \pm O({\varepsilon }^3) . \end{aligned}$$

Taking squares, we get (35) and (36), but mind that this is only for the special case in which the apex projects orthogonally to the circumcenter of the base. To prove the bounds in the general case, we recall that Lemma 4.3 asserts that the projection of *a* onto ${\mathrm{aff\,}{Q}}$ is at most $O(\Delta ^3)$ units of length from $z_Q$. Hence, we get an additional error term of $O(\Delta ^3)$ in all the above equations, but this does not change any of the bounds as stated. $\square$

Note that $D_S$ is the minimum of the $D_{Q,S}$, over all facets *Q* of *S*. Hence, Lemma 4.4 proves Hypothesis II in the case in which *S* has no short edges.

#### Inductive Step (Bi-pyramid Case)

The second kind of inductive step—from $(\ell , j-1)$ to $(\ell , j)$—makes use of a distance function between affine subspaces of ${{\mathbb R}}^d$. In our case, the function measures the distance from a *p*-plane to a $(d-1)$-plane, which is linear provided the distance is taken with a sign that is different on the two sides of the hyperplane.

##### Lemma 4.5

(Bi-pyramid Step) Let $d = 2k+1$, $\Delta > 0$ sufficiently small, $A = A_d (n,\Delta ) \subseteq {{\mathbb R}}^d$, and ${\varepsilon }= {\varepsilon }(n,\Delta )$. Furthermore, let $S \in {\textrm{Del}{({A})}}$, with $\ell = {{\ell }{({S})}}$ and $j = {{j}{({S})}} \ge 0$, and let *a* and $a''$ be the endpoints of a short edge. Assuming $Q = S-a''$ and $Q'' = S-a$ satisfy Hypotheses I and II, we have

47 $$\begin{aligned} D_{Q,S}^2&= \frac{1}{(\ell +1)^2} {\varepsilon }^2 \pm O({\varepsilon }^3); \end{aligned}$$

48 $$\begin{aligned} R_S^2&= R_\ell ^2 + \frac{j+1}{(\ell +1)^2} {\varepsilon }^2 \pm O({\varepsilon }^3); \end{aligned}$$

##### Proof

By construction, ${{\ell }{({Q})}} = {{\ell }{({Q''})}} = \ell$, ${{j}{({Q})}} = {{j}{({Q''})}} = j-1$, and $(a, Q-a)$ and $(a'', Q''-a'')$ are pyramids. We write $P = Q-a = Q''-a''$ for the common base, which has ${{\ell }{({P})}} = \ell -1$ and ${{j}{({P})}} = j-1$. Let *M* be the bisector of *a* and $a''$. It intersects the short edge orthogonally at its midpoint. Letting $\psi :{\mathrm{aff\,}{Q}} \rightarrow {{\mathbb R}}$ map each point of ${\mathrm{aff\,}{Q}}$ to its distance from the nearest point on *M*, we have $\psi (a) = {\varepsilon }$ and, by Lemma 4.3, $\psi (b) = O(\Delta ^3)$, for each vertex *b* of *P*. Let $a'$ be the projection of *a* onto ${\mathrm{aff\,}{P}}$. By Hypothesis II and Lemma 4.3, $a'$ is closer to $z_P$ than the radius of the largest ball centered at $z_P$ which is contained in *P*. Hence, $a' \in P$, so $\psi (a') =O(\Delta ^3)$ by the linearity of the signed version of $\psi$. To compute the gradient of this linear function, we recall Lemma 4.4, which asserts

49 $$\begin{aligned} H_{P,Q}^2&= H_\ell ^2 - \frac{j}{\ell ^2} {\varepsilon }^2 \pm O({\varepsilon }^3) ; \end{aligned}$$

50 $$\begin{aligned} D_{P,Q}^2&= D_\ell ^2 - \frac{(2\ell +1) j}{\ell ^2 (\ell +1)^2} {\varepsilon }^2 \pm O({\varepsilon }^3) . \end{aligned}$$

We compute the length of the gradient as the ratio of the difference in function value, which is ${\varepsilon }$, and the distance between the points, as given in (49). Using (13) to simplify the expression, we first get the length of the gradient of $\psi$ and second the value at the circumcenter of *Q*:

51 $$\begin{aligned} {\Vert {\nabla \psi }\Vert }&= \frac{{\varepsilon }}{H_{P,Q}} \pm O(\Delta ^3) = \frac{{\varepsilon }}{H_\ell } \pm O({\varepsilon }^3); \end{aligned}$$

52 $$\begin{aligned} \psi (z_Q)&= \frac{D_\ell \cdot {\varepsilon }}{H_\ell } \pm O({\varepsilon }^3) = \frac{{\varepsilon }}{\ell +1} \pm O({\varepsilon }^3), \end{aligned}$$

in which we exploit that (50) gives a bound on the distance of the circumcenter from *P*, and we use (28) to get the right-hand side. Hence, ${\Vert {z_Q}-{z_S}\Vert } = {\varepsilon }/(\ell +1) \pm O({\varepsilon }^3)$, which implies

53 $$\begin{aligned} D_{Q,S}^2&= \frac{1}{(\ell +1)^2} {\varepsilon }^2 \pm O({\varepsilon }^3) ; \end{aligned}$$

54 $$\begin{aligned} R_S^2&= R_Q^2 + \frac{1}{(\ell +1)^2} {\varepsilon }^2 \pm O({\varepsilon }^3) = R_\ell ^2 + \frac{j+1}{(\ell +1)^2} {\varepsilon }^2 \pm O({\varepsilon }^3), \end{aligned}$$

where we used the inductive assumption for $R_Q^2$ to obtain the bounds for $R_S^2$. This proves (47) and (48). $\square$

This completes the inductive argument, establishing Hypotheses I and II. In particular, the bounds furnished for the $D_{Q,S}$ imply the required bound for $D_S$, which is the minimum over all facets *Q* of *S*.

### All Simplices are Critical

The above analysis implies that for sufficiently small $\Delta > 0$ the circumcenter of every simplex in ${\textrm{Del}{({A})}}$ is contained in the interior of the simplex. This is half of the proof that all simplices in ${\textrm{Del}{({A})}}$ are critical. The second half of the proof is not difficult.

#### Corollary 4.6

(All Critical in ${{\mathbb R}}^{2k+1}$) Let $d = 2k+1$, $n \ge 2$, $\Delta > 0$ sufficiently small, and $A = A_d (n,\Delta ) \subseteq {{\mathbb R}}^d$. Then every simplex in ${\textrm{Del}{({A})}}$ is a critical simplex of ${\textrm{Rad}}:{\textrm{Del}{({A})}} \rightarrow {{\mathbb R}}$.

#### Proof

A simplex $S \in {\textrm{Del}{({A})}}$ is a critical simplex of ${\textrm{Rad}}$ iff it contains the circumcenter in its interior, and the $(d-1)$-sphere centered at the circumcenter and passing through the vertices of *S* does not enclose or pass through any of the other points of *A*. By Hypothesis II, the first condition holds. To derive a contradiction, assume the second condition fails for $S \in {\textrm{Del}{({A})}}$. In other words, there is a point, $b \in A$, that is not a vertex of *S* but it is enclosed by or lies on the said $(d-1)$-sphere. If $\mathrm{dim\,}{S} = d$, then the $(d-1)$-sphere intersects each circle in two points; that is: each ${{C}_{\ell }}$ for $0 \le \ell \le k$. But in this case, there is no possibility for another point to interfere, so we may assume $\mathrm{dim\,}{S} < d$.

Since a sphere and a circle intersect in at most two points, we may assume that *b* lies on a circle not touched by *S*, or that *b* neighbors a vertex of *S* along its circle, and it is the only vertex of *S* on this circle. Then we can add *b* as a new vertex to get a simplex *T* with $\mathrm{dim\,}{T} = \mathrm{dim\,}{S} + 1$. This simplex also belongs to ${\textrm{Del}{({A})}}$ and, by construction, its circumcenter lies beyond the face *S* as seen from the new vertex of *T*. In other words, the circumcenter does not lie in its interior, which contradicts Hypothesis II. $\square$

### Counting the Cycles

The final counting argument is similar to the one for even dimensions, with a few crucial differences. Instead of congruent simplices, we have almost congruent simplices in odd dimensions, but they are similar enough to be separated by their circumradii.

#### Corollary 4.7

(Ordering of Radii in ${{\mathbb R}}^{2k+1}$) Let $d = 2k+1$, $n \ge 2$, $\Delta > 0$ sufficiently small, $A = A_{2k+1}(n,\Delta ) \subseteq {{\mathbb R}}^{2k+1}$, and ${\textrm{Rad}}:{\textrm{Del}{({A})}} \rightarrow {{\mathbb R}}$ the radius function. Then the circumradii of two simplices, $S, T \in {\textrm{Del}{({A})}}$, satisfy ${\textrm{Rad}}(S) < {\textrm{Rad}}(T)$ if ${{\ell }{({S})}} < {{\ell }{({T})}}$, or ${{\ell }{({S})}} = {{\ell }{({T})}}$ and ${{j}{({S})}} < {{j}{({T})}}$.

#### Proof

By Corollary 4.6, the circumradii are the values of the simplices under the radius function, and by Hypothesis I, the circumradii are segregated into groups according to the number of touched circles and the number of short edges. It follows that the values of ${\textrm{Rad}}$ are segregated the same way. $\square$

Let $\varrho _{\ell ,j}$ be a threshold so that ${\textrm{Rad}}(S)< \varrho _{\ell ,j} < {\textrm{Rad}}(T)$ for all simplices *S* and *T* that satisfy ${{\ell }{({S})}} < \ell$ or ${{\ell }{({S})}} = \ell$ and ${{j}{({S})}} \le j$, and ${{\ell }{({T})}} > \ell$ or ${{\ell }{({T})}} = \ell$ and ${{j}{({T})}} > j$. For $0 \le \ell \le k$ and $-1 \le j \le k$, we are interested in three kinds of these thresholds:

- $\varrho _{\ell -1,\ell -1}$, which separates the simplices that touch at most $\ell$ circles from those that touch at least $\ell +1$ circles;
- $\varrho _{\ell ,-1}$, which separates the $\ell$-simplices without short edges from the other simplices that touch the same number of circles;
- $\varrho _{k,j}$, which separates the $(k+j+1)$-simplices that touch all $k+1$ circles from the $(k+j+2)$-simplices that touch all $k+1$ circles.

We begin by studying the Alpha complexes defined by the first type of thresholds, ${\mathcal {A}_{{\ell -1},{\ell -1}}} = {\textrm{Rad}}^{-1} [0, \varrho _{\ell -1,\ell -1}]$.

#### Lemma 4.8

(Constant Homology in ${{\mathbb R}}^{2k+1}$) Let $d = 2k+1$ be a constant, $A = A_{d} (n, \Delta ) \subseteq {{\mathbb R}}^{2k+1}$, and $1 \le \ell \le k$. Then ${\beta }_{p}{({\mathcal {A}_{{\ell -1},{\ell -1}}})} = O(1)$ for every *p*.

#### Proof

Pick $\ell$ of the $k+1$ circles used in the construction of *A*, let $A' \subseteq A$ be the points on these $\ell$ circles, and note that the full subcomplex of ${\textrm{Del}{({A})}}$ with vertices in $A'$ has no non-trivial (reduced) homology. We may collapse this full subcomplex to a single $(\ell -1)$-simplex. ${\mathcal {A}_{{\ell -1},{\ell -1}}}$ is the union of $$\left( {\begin{array}{c}k+1\\ \ell \end{array}}\right) $$ such full subcomplexes of ${\textrm{Del}{({A})}}$, one for each choice of $\ell$ circles. The intersections of these subcomplexes are of the same type, namely induced subcomplexes of ${\textrm{Del}{({A})}}$ for the points on $\ell$ or fewer of the circles. Hence, ${\mathcal {A}_{{\ell -1},{\ell -1}}}$ has the homotopy type of the complete $(\ell -1)$-dimensional simplicial complex with $k+1$ vertices. Its $(\ell -1)$-st homology group is the only non-trivial homology group, and its rank is a constant independent of *n* and $\Delta$, as required. $\square$

To prove relation (25) of Theorem 4.1, we second consider the Alpha complexes defined by the second type of thresholds, ${\mathcal {A}_{{\ell },{-1}}} = {\textrm{Rad}}^{-1} [0, \varrho _{\ell ,-1}]$. This complex is ${\mathcal {A}_{{\ell -1},{\ell -1}}}$ together with all $\ell$-simplices without short edges. By Lemma 4.8, only a constant number of them give death to $(\ell -1)$-cycles, while all others give birth to $\ell$-cycles. This implies that the rank of the $\ell$-th homology group of ${\mathcal {A}_{{\ell },{-1}}}$ is the number of $\ell$-simplices without short edges minus a constant, which is $$\left( {\begin{array}{c}k+1\\ \ell +1\end{array}}\right) (n+1)^{\ell +1} \pm O(1)$$. This construction works for $0 \le \ell \le k$, which implies relation (25).

To prove relation (26) inductively, we third consider the Alpha complexes defined by the third type of thresholds, ${\mathcal {A}_{{k},{j}}} = {\textrm{Rad}}^{-1} [0, \varrho _{k,j}]$, for $0 \le j \le k$. The induction hypothesis is

55 $$\begin{aligned} {\beta }_{p}{({\mathcal {A}_{{k},{p-k-1}}})}&= \genfrac(){0.0pt}1{k}{p-k} \cdot (n+1)^{k+1} \pm O(n^k) , \end{aligned}$$

and we use the case $p=k$ of relation (25) as the induction basis. The difference between ${\mathcal {A}_{{k},{p-k-1}}}$ and ${\mathcal {A}_{{k},{p-k}}}$ are the $(p+1)$-simplices with $p-k+1$ short edges. Their number is

56 $$\begin{aligned} \genfrac(){0.0pt}1{k+1}{p-k+1} \cdot (n+1)^{2k-p} n^{p-k+1}&= \genfrac(){0.0pt}1{k+1}{p-k+1} \cdot (n+1)^{k+1} \pm O(n^k) , \end{aligned}$$

This number divides up into the ones that give death and the remaining ones that give birth. Since $$\left( {\begin{array}{c}k+1\\ p-k+1\end{array}}\right) - \left( {\begin{array}{c}k\\ p-k\end{array}}\right) = \left( {\begin{array}{c}k\\ p-k+1\end{array}}\right) $$, this implies

57 $$\begin{aligned} {\beta }_{p+1}{({\mathcal {A}_{{k},{p-k}}})}&= \genfrac(){0.0pt}1{k}{p-k+1} \cdot (n+1)^{k+1} \pm O(n^k) , \end{aligned}$$

as needed to finish the inductive argument.

### Voids in Even Dimensions

We return to the one case in $d = 2k$ dimensions that is not covered by the construction in Section 2, namely the $(2k-1)$-st Betti number. It counts the top-dimensional holes, which we refer to as *voids*. Notwithstanding that the construction in Section 2 does not provide any voids, Theorem 2.1 claims the existence of $N = k(n+1) + 2$ points in ${{\mathbb R}}^{2k}$ and a radius such that ${\beta }_{2k-1}{} = n^k \pm O(n^{k-1})$.

The set of *N* points whose Čech complex has that many voids is a straightforward modification of the construction in $2k-1$ dimensions: place $A = A_{2k-1} (n, \Delta )$ in the $(2k-1)$-dimensional hyperplane $x_{2k} = 0$ in ${{\mathbb R}}^{2k}$. Every $(2k-2)$-cycle—which corresponds to a void in $2k-1$ dimensions—is now a pore in the hyperplane that connects the two half-spaces. In the odd-dimensional construction, all pores arise when the radius is roughly $R_{k-1} \ge \frac{1}{2}$, and they are located in a small neighborhood of the origin. By choosing $\Delta > 0$ sufficiently small, we can make this neighborhood arbitrarily small. It is thus easy to add two points, one on each side of the hyperplane, such that their balls close the pores from both sides and turn them into voids in ${{\mathbb R}}^{2k}$. More formally, the two points doubly suspend each $(2k-2)$-cycle into a $(2k-1)$-cycle. Hence, Theorem 4.1 for $d=2k-1$ and $p=2k-2$, which gives ${\beta }_{p}{} = (n+1)^k \pm O(n^{k-1})$, provides the missing case in the proof of Theorem 2.1.

## Discussion

In this paper, we give asymptotically tight bounds for the maximum *p*-th Betti number of the Čech complex of *N* points in ${{\mathbb R}}^d$. These bounds also apply to the related Alpha complex and the dual union of equal-size balls in ${{\mathbb R}}^d$. They do not apply to the Vietoris–Rips complex, which is the flag complex that shares the 1-skeleton with the Čech complex for the same data. In other words, the Vietoris–Rips complex can be constructed by adding all 2- and higher-dimensional simplices whose complete set of edges belongs the 1-skeleton of the Čech complex. This implies , since adding a triangle may lower but cannot increase the first Betti number.

As proved by Goff [15], the 1-st Betti number of the Vietoris–Rips complex of *N* points is *O*(*N*), for all radii and in all dimensions, so also in ${{\mathbb R}}^3$. Compare this with the quadratic lower bound for Čech complexes proved in this paper. This implies that the first homology group of this Čech complex has a basis in which most generators are tri-gons; that is: the three edges of a triangle. The circumradius of a tri-gon is less than $\sqrt{2}$ times the half-length of its longest edge, which implies that most of the $\Theta (N^2)$ generators exist only for a short range of radii. In the language of persistent homology [9], most points in the 1-dimensional persistence diagram represent 1-cycles with small persistence. Similarly, the 2-nd Betti number of a Vietoris–Rips complex in ${{\mathbb R}}^3$ is $o(N^2)$ [15], compared to that of a Čech complex, which can be $\Theta (N^2)$. Hence, most points in the corresponding persistence diagram represent 2-cycles with small persistence.
